# Headache Profile of Patients Attending Otorhinolaryngology Clinics: Navigating Diagnostic Challenges

**DOI:** 10.1055/s-0046-1818584

**Published:** 2026-04-01

**Authors:** Thripthi Rai, Keshava K. Pai, Meera Niranjan Khadilkar, Vijendra Shenoy

**Affiliations:** 1 Department of Otorhinolaryngology - Head and Neck, Kasturba Medical College Mangalore, Manipal Academy of Higher Education, Manipal, India; 2 Department of Psychiatry, Kasturba Medical College Mangalore, Manipal Academy of Higher Education, Manipal, India

**Keywords:** headache, headache disorders, health, migraine disorders, otolaryngology, sinusitis

## Abstract

**Introduction:**

Headache is a common complaint among otolaryngology patients, often presenting with an apparent diagnosis of *secondary headache* due to sinusitis. However, associated symptoms may also be linked to *primary headache* disorders such as migraines. The overlapping symptomatology of various headache causes poses a diagnostic challenge. The present study aims to evaluate the headache profiles of patients visiting the otolaryngology department.

**Objective:**

The present study aims to evaluate the headache profiles of patients visiting the otolaryngology department.

**Methods:**

A cross-sectional study was conducted on 128 otolaryngology patients presenting with headaches. The headaches were categorized according to the International Classification of Headache Disorders, third edition (ICHD-3), based on detailed history, examination, nasal endoscopy, and imaging studies.

**Results:**

Primary headaches were diagnosed in 45.31% of the cases, while 54.69% presented secondary headaches. Sinusitis was the most common diagnosis, found in 40.63% of the patients. Various causes of primary and secondary headaches were correlated and were found to be statistically significant ( *p* < 0.001 each). Statistical significance was observed when correlating primary headache with laterality ( *p* < 0.001). The correlation of secondary headache with laterality and age range also showed statistical significance ( *p* < 0.001 and 0.035 respectively).

**Conclusion:**

Sinonasal symptoms may accompany headaches, with or without a direct causal link. The accurate diagnosis and treatment of headache disorders requires a thorough understanding of the latest guidelines and a multidisciplinary approach. This comprehensive strategy is essential to provide high-quality healthcare and significantly improve the quality of life of individuals suffering from this common and often debilitating condition.

## Introduction

Headache disorders impose a substantial burden on public health, affecting ∼ 47% of adults worldwide, with a significant socioeconomic impact leading to missed workdays and decreased productivity. 1 In India, an estimated 34% of the population experiences tension-type headache (TTH), while 28% suffer from migraine, leading to a considerable impact on quality of life and healthcare expenditures. 2 In the otolaryngology patient population, headache disorders are particularly prevalent, often presenting with overlapping symptoms of sinonasal disease. Studies 3 have shown that up to 60% of patients with chronic rhinosinusitis (CRS) experience comorbid headache disorders. However, differentiating between primary and secondary headaches in this population poses a significant diagnostic challenge due to complex symptom overlap. Primary headache disorders, such as migraines, often present symptoms like sinonasal disease; conversely, secondary headaches caused by sinonasal disease can manifest with symptoms resembling primary headache disorders. This diagnostic conundrum requires a multidisciplinary approach, involving general physicians, neurologists, ophthalmologists, otolaryngologists, and psychiatrists. Despite the high prevalence and burden of headache disorders in India, there is a paucity of literature on the evaluation and management of cases of headache presenting to otolaryngology clinics, highlighting the need for studies like the present one, which aims to evaluate the headache profile of patients visiting the otolaryngology department, to provide valuable insights into the epidemiology, diagnosis, and management of headache disorders in this population.

## Methods

After obtaining clearance from the institutional Ethics Committee, a cross-sectional study was conducted throughout 1 year on 128 patients in the age range of 18 to 65 years presenting to the otolaryngology department with headache complaints at a tertiary care hospital in a coastal city of Southern India. Patients with incomplete diagnostic workup and cases in which a definitive diagnosis could not be established were excluded. Written informed consent was obtained from all participants. Following thorough physical and otolaryngological examinations, diagnostic nasal endoscopy (DNE) was performed in all cases by the first author (TR). Computed tomography (CT) scans of the paranasal sinuses and brain magnetic resonance imaging (MRI) scans were performed when indicated. A single radiologist performed all the radiological reporting. The type of headache was classified according to the International Classification of Headache Disorders, third edition (ICHD-3), 4 based on detailed history, examinations, and proper investigations. When no rhinogenic or primary cause for the headache was found, the patient was referred to a psychiatric, ophthalmological, or/and neurological consultation. The diagnoses of secondary headaches established by these departments was recorded. Data were statistically analyzed using the Jamovi software, version 1.8.1 (open source), with the results expressed as proportions and summary measures. The type of headache was correlated through the Chi-squared test. Primary and secondary headaches were individually correlated with age, gender, laterality, and duration using the Chi-squared and Kruskal-Wallis tests, with a *p* -value below 0.05 considered statistically significant, and below 0.001, highly significant.

## Results

A total of 128 patients with headache were studied ( Table 1 ). Their mean and median ages were of 36 and 35 years respectively, ranging from 18 to 65 years. The sample included 44 (34.38%) male and 84 (65.63%) female subjects. Most patients (29.69&) were aged between 26 and 35 years, and 84 (65.63%) patients had headaches for longer than 3 months. Bilateral headache was noted in 84.38% of the cases; headache was diffuse in most of the patients (67.19%). Nasal obstruction was present in 48 (37.5%) of the patients, and nausea, in 20 (15.63%). No history of snoring or sleep disturbances was noted in any patient, thus ruling out sleep apnoea-associated headache. History of head trauma was noted in 3 (2.34%) cases, and nasal trauma in 1 (0.78%). The ear examination was unremarkable for all patients. Revealed deviated nasal septum in 96 (75%) of the cases, 6 (4.69%) of them with gross deviation in contact with the lateral nasal wall. Pale mucosa was noted in 28 (21.88%) of the cases, 8.59% presented ethmoidal nasal polyps, and 4 (3.13%), antrochoanal polyps. Temporal artery palpation was within normal limits in all (100%) patients. Tenderness over the cervical or temporomandibular joints and movement limitation was absent in all (100%) patients. Auscultation over the skull, carotid and vertebral vessels was normal in all (100%) patients. A total of 10 (7.81%) patients were referred to other departments for an evaluation of non-rhinogenic causes of secondary headache. Overall, 58 (45.31%) patients were diagnosed with primary headache, while 70 (54.69%) were diagnosed with secondary headache. Most patients (52; 40.63%) were diagnosed with sinusitis, 16 (30.77%) of whom presented associated sinonasal polyposis. Statistical significance was observed when correlating primary headache with laterality and secondary headache with laterality and age ( Tables 2 3 ).

**Table 1:** Types of headache among the study sample

Primary headache	N (%)	Chi-squared goodness of fit test	Degrees of freedom	*p*
Migraine	40 (68.97%)	38.2	2	< 0.001*
Tension	16 (27.59%)			
Cluster	2 (3.45%)			
**Secondary headache**		130	4	< 0.001*
Sinusitis	8 (11.43%)			
Refractive	52 (74.29%)			
Somatoform	4 (5.71%)			
Hypertension	4 (5.71%)			
Sinusitis	2 (2.86%)			

Note: *Statistically significant.

**Table 2:** Primary headache and correlation with various parameters

	Primary headache				Chi-squared goodness of fit test			Kruskal-Wallis test			
	Migraine	Tension headache	Cluster headache	Total	Chi-squared	Degrees of freedom	*p*	Chi-squared	Degrees of freedom	*p*	Eta-squared
**Age group (in years)**											
18–25	12	2	0	14	4.5	6	0.61	2.7	3	0.43	0.048
26–35	16	8	2	26							
36–45	8	4	0	12							
46–55	4	2	0	6							
Total	40	16	2	58							
**Gender**											
Male	6	6	0	12	4.1	2	0.13	0.89	1	0.34	0.016
Female	34	10	2	46							
**Laterality**											
Unilateral	8	0	2	10	13	2	0.001*	0.17	1	0.68	0.003
Bilateral	32	16	0	48							
**Duration of headache**											
≤ 3 months	8	6	0	14	2.6	2	0.28	0.89	1	0.34	0.016
> 3 months	32	10	2	44							

**Note:** *Statistically significant.

**Table 3:** Secondary headache and correlation with various parameters

	Secondary headache						Chi-squared goodness of fit test			Kruskal-Wallis test			
	Non-sinus Rhinogenic	Sinusitis	Refractive error	Somatoform	Hypertension	Total	Chi-squared	Degrees of freedom	*p*	Chi-squared	Degrees of freedom	*p*	Eta-squared
**Age range**													
18–25	0	14	0	0	0	14	42.1	16	< 0.001*	3.6	4	0.463	0.0522
26–35	2	8	2	0	0	12							
36–45	4	6	2	4	0	16							
46–55	2	16	0	0	0	18							
56–65	0	8	0	0	2	10							
**Gender**													
Male	4	24	0	2	0	30	4.98	4	0.289	1.77	1	0.183	0.0257
Female	4	28	4	2	2	40							
**Laterality**													
Unilateral	4	6	0	0	0	10	10.3	4	0.035*	8.09	1	0.004	0.117
Bilateral	4	46	4	4	2	60							
**Duration of headache**													
≤ 3 months	6	20	2	2	0	30	5.45	4	0.244	1.94	1	0.164	0.0281
> 3 months	2	32	2	2	2	40							

Note: *Statistically significant.

## Discussion

Headaches are a worldwide infirmity, distressing almost everyone at least once in their lifetimes, and they have remained a diagnostic and therapeutic challenge for doctors since time immemorial. 5 Primary headaches are idiopathic conditions without an established origin, such as migraine, TTH, and cluster headache, while secondary headaches occur due to another pathological process in the nose or eyes, or due to vascular or psychological issues. 6 Studies have shown that 49% of patients visiting otolaryngology clinics due to sinus headache presented migraine, while only 13% presented migraine alone. 7 A history of headache was seen to be more frequent in patients with CRS than in non-CRS cases. Chronic rhinosinusitis has been reported 7 as a factor in the deteriorating of the course of migraine, possibly making it more chronic or refractory. Thus, the association of CRS and migraine remains unclear. In the current study, 58 (45.31%) patients were diagnosed with primary headaches, while 70 (54.69%) were diagnosed with secondary headaches.

Nearly 30% of the patients in the study were aged between 26 and 35 years, with migraines being the most common cause 16 (42.11%) of headaches in this age group, followed by TTH 8 (21.05%). Contrary to most long-lasting conditions, migraine typically affects young or middle-aged individuals, as found in a study by Peters. 8 In the present study, migraines affected 16 (40%) patients in the age group from 26 to 35 years, and 12 (30%) of the patients aged between 18 and 25 years. This finding could be linked to a stressful lifestyle, prolonged working hours, obesity, delayed or missed meals, artificial sweeteners, and caffeine intake. 8 The occurrence of migraine reduces with age, 8 as was also observed in the current study. Migraines were significantly more frequent 34 (85%) in women in the present study. Fluctuating hormonal levels, particularly estrogen, and the nature of the menstrual cycle may incite migraine attacks. 9 In total, 30 (75%) of the 40 migraine cases presented with migraine-related symptoms such as nausea, aura, and photophobia in the current study. Of these, 29 patients (96.67%) were women. A plausible explanation is that menstruation-related migraine is longer-lasting, more intense, more likely to present with cutaneous allodynia, and more resistant to medication. 9 Most participants 84 (65.63%) in the present study were women; however, no statistical significance was observed between gender and headache etiology ( *p* -values 0.13 and 0.289 for primary and secondary headaches respectively).

The most predominant type of primary headache in the general population is TTH, 10 contrary to the findings of the present study. It is a mild-to-moderate headache without debilitating migraine symptoms. Due to its less severe nature, TTH patients are less likely to seek medical consultation than patients with migraine; 10 this is a probable reason for the lesser magnitude of TTH in the present study. Like migraine, TTH was most frequent in the age group from 26 to 35 years, with 8 (50%) cases in the present study.

Cluster headaches were observed in 2 patients (1.56%), both women in their fourth decade of life, with episodes of frontotemporal headaches. The pain was unilateral, as noted in 18 other patients, including those with migraines (8 cases), sinusitis (6 cases), and septal deviation (4 cases). Though migraine is typically characterized by unilateral headache, we found only 8 (6.25%) such cases; a diagnosis of migraine was established based on the other criteria. 4 The comparison of laterality and cause of headache showed statistical significance ( *p* -values of 0.001 and 0.035 for primary and secondary headaches respectively).

Patients often visit the otolaryngologist with a self-diagnosis of *sinus headache* . 11 This term is frequently used by patients, primary care physicians, the media, and advertisers, but it is considered inaccurate by otolaryngologists and neurologists, leading to inadequate headache management. Patients also typically complain of facial pain or pressure, a symptom traditionally associated with sinusitis. *Rhinogenic headache* is a more precise term if the symptoms arise from the nasal region. 5 The otolaryngologist plays a critical role in accurately diagnosing and managing these headaches. The intricacy of diagnosing headaches in otolaryngology patients, combined with practical restrictions in primary care settings, often leads to frequent misdiagnoses and unsuitable treatments. Making the proper diagnosis of rhinogenic headache requires complete history and physical examination, including DNE and imaging studies, if necessary. 11 In the current study, 60 (46.88%) of the 128 patients were found to have either sinus or nasal pathology; 52 (83.33%) of these cases presented sinusitis, while 8 (16.67%), other causes of rhinogenic headaches, such as deviated nasal septum and nasal vestibulitis. There is a significant overlap of symptoms between migraine and sinusitis due to the common irritation of the trigeminal nerve, which supplies the paranasal sinuses and meninges. A history of nasal obstruction and discharge can also be part of the clinical presentation of migraine due to autonomic nerve stimulation. Therefore, these symptoms do not necessarily indicate sinusitis. 12 In the present study, associated nasal and aural symptoms included nasal obstruction 48 (37.5%), nasal discharge 10 (7.81%), giddiness 8 (6.25%), and decreased smell perception or epistaxis 4 (3.13%). Sinusitis was confirmed through DNE and CT scans of the paranasal sinus. Rhinogenic contact point headache is pain from a contact point between the septum and lateral nasal wall due to a septal deviation or spur, concha bullosa or ethmoidal bulla. 13 In the present study, although a deviated nasal septum was found in 69 (53.9%) of the patients, gross deviation was only observed in 6 (4.69%) cases, septal spur, in 20 (15.63%), and concha bullosa, in 17 (13.28%). Rhinogenic headaches were most frequent 18 (30%) in the age group from 46 to 55 year, followed by the group from 18 to 25 years 14 (23.33%), and were slightly more frequent in women (gender ratio: 0.88).

The ICHD-3 categorizes headaches lasting beyond 3 months as chronic headaches. 4 In the current study, 100 (78.13%) of the 128 patients presented chronic headaches, with some experiencing symptoms for as long as 25 years. Sinusitis was the most common cause of chronicity in 44 (44%) of these 100 cases, followed by migraine in 32 (32%) cases. No statistical significance ( *p* -values of 0.28 and 0.244 for primary and secondary headaches respectively) was found between the duration and cause of headaches. Patients with headaches often take over-the-counter drugs or consult with pain specialists such as general practitioners, chiropractors, physical therapists, and dentists; 10 this may explain the long-standing history of headache in our patients.

No otolaryngological or primary cause for the headaches was observed in 10 (7.81%) patients. Ophthalmological and psychiatric evaluations were performed in 8 (6.25%) patients each, with 4 diagnosed with refractive errors and somatoform disorders respectively. Although 4 (31.25%) patients presented a history of trauma, no conclusive findings were obtained on imaging. In total, 8 (6.25%) patients attended neurological consultations,; the MRI scans showed no intracranial abnormalities in 6 of these cases. A total of 2 (1.56%) patients were diagnosed with hypertensive crisis-related headaches with mild ischemic changes on MRI; both were women in their seventh decade of life. Not all neurologists have a keen interest in headaches, and not all physicians offering outstanding headache care are neurologists. Otolaryngologists play a vital role in enhancing patient care, even when a positive imaging study is genuinely negative. 14 Our aim as otolaryngologists was to assess, screen, and treat patients presenting with headaches based on the most recent evidence-based criteria and protocols for the common causes of headache. 11 Despite this being an issue routinely encountered in the otolaryngology practice, few studies have been conducted 15 on the typification of and screening for headache in the vast Indian population, to the best of our understanding. However, the current study does not discuss the role of various treatment strategies in the management of this common affliction, leaving room for future research to explore long-term management outcomes and novel diagnostic or treatment approaches. Another limitation of the present study is that there may have been a selection bias, since only patients visiting the Otolaryngology Department were included, thus leading to an overrepresentation of patients with secondary headache due to sinonasal disease. Patients excluded from the study due to incomplete diagnostic workup or inability to establish a conclusive diagnosis may also add to the selection bias, reduced generalizability, loss of valuable data, and misclassification bias.

## Conclusion

Headache is a prevalent complaint in the otolaryngology practice, often accompanied by sinonasal symptoms that may or may not have a direct causal link. The complexity of headache disorders and the overlap of symptoms often lead to unnecessary investigations due to insufficient knowledge of the diagnostic criteria, classifications, and treatment protocols. The findings of the current study, highlighting the high prevalence of secondary headaches, particularly sinusitis, and the importance of correlating causes with age, gender, and laterality, underscore the need for a comprehensive understanding of headache disorders. The takeaway may be summarized as an emphasis on accurate diagnosis by differentiating between primary and secondary headaches; identifying at-risk groups by understanding how age and gender influence headache; improving patient outcome by addressing both migraines and sinusitis when present simultaneously; preventive measures by modifying lifestyle; and enhancing treatment efficacy for women by considering the hormonal influences. These insights underscore the need for a tailored and collaborative approach to headache management. Adherence to the latest guidelines and a multidisciplinary team approach are crucial for the accurate diagnosis and effective management of headache disorders. This approach is essential to provide high-quality healthcare and significantly improve the quality of life of individuals suffering from this common and often debilitating condition.
